# A System of Practical Surgery

**Published:** 1853-05

**Authors:** 


ART. XI.—*A System of Practical Surgery, By* WILLIAM FERGUSSON, F. R. S., Professor of Surgery in King's College, London; Surgeon to King's College Hospital; and Surgeon to H. R. H. Prince Albert. Fourth American from the third and enlarged London edition, with three hundred and ninety-three illustrations. Philadelphia, Blanchard & Lea, 1853: 8vo., pp. 621.

On all sides, the System of Surgery by Professor Fergusson is admitted to be one of the most complete and valuable ever given to the public. The experience of the author, extending over thirty years, enables him to speak with confidence on all the points of practical surgery. His work is, therefore, no production of the closet, but the fruits of his own observation in hospitals and his large private practice. We do not remember a volume in the study of which the reader will find more aid from the illustrations; the wood cuts are very numerous, and are exceedingly well executed. Beginning with "Instruments, incisions, dissections and operations," everything is exhibited to the eye that is susceptible of demonstration, and the student may read this chapter with the feeling that he comprehends every operative process described.

Prof. F. speaks warmly of anæsthesia in operative surgery, as "a vast and important change," by which, "instead of wild outcries or stifled screams and groans," the patient may be made to lie as quietly as if in a calm sleep." He thinks "it is not saying too much when this discovery is characterized as the greatest, in most respects, which has been made in the province of surgery." We agree with him in this opinion; but as there are many practitioners who still hesitate about the propriety of using anæsthetics in surgery, we quote from him on this subject.

"While the custom of rendering patients insensible to pain, during the performance of surgical operations, has rapidly gained an undoubted and sure position, there have been various quesions and drawbacks regarding it, many of which are, even at the present day, of great practical importance. Several deaths have occurred, to all appearance, directly from the anæsthetic agents, or the mode of their administration, and there has been much discussion on both these points. In the early application of ether, a complicated apparatus was used, but soon it was found that by holding over the patient's nostrils and mouth, a handkerchief, napkin, or piece of sponge, moistened with a few drops of the anæsthetic, the desired effect was speedily produced, and, to this day, perhaps the majority of those who use the agent are content with this mode of administering it. From the commencement it has been the custom of most surgeons in large operating practice, to have the agent administered by some one whose sole attention might be given to its physiological effects on the patient, during the performance of the operation; and among those who have performed this most important duty, there are few so extensiveely experienced as Dr. Snow. This gentleman, who is justly deemed our highest authority on the subject of anæsthesia in surgical operations, is in the habit of using an apparatus, as he is of opinion that there is less danger and more certainty thereby than with a napkin. There are different views on this subject, however, which need not be discussed here, and it may be sufficient to say that in whatever way the agent is called into use, its powers are fraught with subtle and imminent danger, unless the greatest caution be taken. For my own part, I think it of little consequence by what means the agent is administered, provided there be a fair proportion of atmospheric air admitted to the lungs at the same time, or that there be a facility of admitting it, when the influence of the chloroform, or whatever agent is employed, seems too powerful.

"At one time it was supposed that certain states of the constitution, certain conditions of the disease, or affections of certain organs, must preclude anæsthesia in surgical operations, but experience has shown that these fears have all been groundless. The age of a patient forms no objection to its use, for it may be given during the first few weeks of life, or at the latest imaginable dat . Nor does it appear that positive disease of any organ forms an insuperable objection to its use. There are certain circumstances in the state of the constitution which will deter the surgeon from performing any important surgical operation, and in such instances the propriety of anæsthesia need never be discussed; but as a general answer to all questions on this score, I am disposed to say, that in whatever case a sugeon may consider an operation justifiable, the use of chloroform (this is named as the agent in most common use) is equally justifiable. If the operation be of minor importance, and of momentary duration, especially if the patient be courageous, and perhaps averse to the practice, the surgeon need say little on the subject; but in all cases where much and protracted suffering is likely, chloroform should be recommended. It will not only save pain, but avert shock, and, provided the agent be properly administered, it will render the surgeon's duties more easy of accomplishment. There are few operations in which its use is not admissible and advantageous. In operations for cataract, especially by extraction, it is likely to prove injurious; as there is no certainty as to the movements of the eyeball and lid for some time after. I have used chloroform for the removal of tonsils, in young persons, but the operation is difficult, and if the patient will keep steady otherwise, it had better not be sued. In staphyloraphy, the co operation of the patient is so essential that I deem chloroform inadmissible. It has been supposed that there is danger from blood trickling into the larynx and trachea in operations under chloroform. In one instance, in the early days of chloroform, I took some alarm on this score, in a case where blood was trickling from the posterior nares into the trachea and causing cough, but I feel now certain that there was no just cause of apprehension. I have since repeatedly removed both upper and lower jaw, and done many other operations, involving considerable hemorrhage towards the throat, but have never seen any more inconvenience from such a cause than if chloroform had not been used. It has happened repeatedly in former times, that persons have died during the performance of operations. Mental emotion, shock, hemorrhage, have each and all had their influence; but, notwithstanding the various instances in which death has happened under chloroform, it may be doubted if sudden death, during operation, has occurred so frequently since the introduction of anæsthesia, as prior to that date. In my own experience I have twice seen patients under chloroform in great jeopardy—one of these had a protracted operation performed for resection of a joint, and possibly a similar state of prostration might have resulted without the use of this agent. In the other instance, tracheotomy was required for disease of the larynx, and under the chloroform, respiration became so slow and difficult that it was feared the patient would die on the instant, before the operation could be executed. When the trachea was opened he immediately breathed more freely, and speedily rallied from his alarming condition.

"I was certainly more impressed with the danger in this instance than in any other that I had seen; but Dr. Snow, who administered the chloroform, did not participate in my fears. The immediate effect of tracheotomy corroborates the view entertained by Ricord and others, that this proceeding may be resorted to with advantage when tnere seems danger from chloroform. When death has occurred seemingly from this agent it has been immediate, and chloroform leaves the system so speedily, that if the patient has once become conscious I doubt if death could ever be attributed to this cause. Chlofoform has no influence whatever either in retarding or accelerating the healing of a wound. We have no statistical data on this subject, but altogether the impression on my mind is, that a patient is better off with chloroform both under and after an operation than if he were without it. When an operation is contemplated under chloroform, almost the only precaution worthy of being taken is, that food should be avoided for several hours before, as chlorcform on a full stomach is almost certin to induce sickness. So much has been written on the subject of chloroform since its introduction that I deem it unnecessary to say much more regarding it at present, and I trust from what has been stated, that it will be understood that, with the few exceptions above made, and such others as might incidentally arise, the use of this agent is recommended as an invariable accompaniment of all surgical operations involving pain.

"The quantity of chloroform required for different patients is very variable. A few drops may suffice for some, while drachms may be needful for others, and generally the amount will be in proportion to the duration of the operation. Some persons take it kindly, even greedily, others make great objections and resistance to its use, both while conscious and unconscious, but all can be at last so thoroughly overpowered by its influence that, compatible with respiration and circulation, the greatest imaginable quietness can be obtained. There is very little effect on the pulse in such cases; sometimes it is slightly excited at first, and if the dose be very potent, it may become slow and weak. There is greater effect on respiration. In some, in the early stages, the movements are rapid, and there seems an eagerness to fill the lungs with the vapor. In others a tickling cough is excited. In many, the full effect is induced with as slight a perceptible change as if sleep had suddenly come on, but in many others great irregularity in the respiratory movements ensues, and much cerebral excitement is induced. Rapid breathing may happen, as already mentioned, but the reverse may be the case, and the power of inspiration may suddenly seem to have ceased. For many seconds there may be no seeming attempt at respiration, but at last a deep breath is usually taken which makes amends for the previous cessation. The cerebral excitement is evinced by crying, bawling, singing, laughing, incoherent talking, and with many there seems some prevailing idea which calls forth unusual and often great muscular action. Some strike forcibly with the fists, and with no seeming want of skill; others use their lower extremities, and others again make such violent general struggles as necessitate the greatest muscular exertion in those around to keep them steady. Often these violent struggles, and the temporary cessation of respiration fall together, and to those not accustomed to such scenes, it appears as if immediate dissolution were impending; but in all such cases a little more of the vapor induces all that quietude which is characteristic of its full effect for the surgeon's purposes. There are various ways in which it may be decided that this period has arrived. Muscular relaxation is one of the best, but perhaps there is no test equal to that of touching the surface of the eyeball with the point of the finger. If there be any indication of feeling, the proper time has not arrived; if the contrary, then the surgeon may proceed. The agent must be administered during the operation as may seem necessary, and it is best to continue its influence until all risk of pain, at the time of operation or dressing, has passed. In most instances it is highly desirable that the patient should be placed on a bed before awaking from his anæsthetic trance. In many cases much mental and bodily suffering may be avoided by administering the chloroform before the patient is moved from bed. In painful diseases of joints, such as of the knee, there is often great suffering in carrying the patient to the operating table, which may, however, be avoided by using chloroform before he is disturbed. Some patients become conscious in a few minutes after the surgeon has ceased his work, while others remain for a longer period as if in calm sleep. In certain instances headache and general uneasiness are complained of for hours or even days after chloroform; but in the majority of cases the effects are slightly if at all felt when consciousness returns."

In the chapter which succeeds, on "the means and instruments for suppressing hemorrhage," there is the same amplitude of illustration—every procedure is exhibited as well as described. He remarks on the means of arresting that troublesome hemorrhage which sometimes follows operations—"the oozing of blood from veins or arteries:"

"Cold air, solutions of spirits of wine, of tinctures, of turpentine, of various salts, such as the acetate of lead, sulphate of alum, of zinc, or of copper, nitrate of silver, caustic potash, even the actual cautery, have all been used for this purpose. From time to time pretended specifics have been used, also; but with these, as was the case with the famous styptic of Brossard, the agaric, pressure seems invariably to be the main agent: and doubtless it would, if properly applied in a severe hemorrhage, be equally efficient without the aid of the mysterious and empirical supposed styptic. Cold air, or cold water, will generally prove successful in ordinary cases, and the cautery will arrest bleeding from very large vessels; but unless these run in bone, or cannot be commanded in any other way, it is now a-days seldom used for such purposes. Any of the medicated fluids enumerated above, used diluted or otherwise, will prove stypticlal in all ordinary oozings. Sometimes they are merely allowed to come in contact with the surface; on other occasions they are combined with pressure, the latter being applied by means of sponge or lint, kept on by the hand or by a bandage. In all instances, the practitioner should bear in mind the favorable influence of keeping the bleeding part above the level of the heart if possible, nor should he forget the remarkable effects of the acetate of lead when used internally in such cases, as well as the peculiar influence of digitalis over the heart's action.

"In many instances the surgeon is obliged to trust to one or the other of these means; but I need scarcely repeat, that when the bleeding is profuse, and when ligatures can be used with propriety, they should generally be preferred."

On the subject of abscess Prof. Fergusson remarks:

"It is, I imagine, too much the custom to allow matter to be discharged naturally. In most parts of the body, if the suppuration be deep seated, the matter may extend widely, and do much harm by the separation of textures, ere it can reach the surface; and even when it does so, and is discharged through some small opening, it rarely happens that the interior of the abscess closes entirely; a discharge continues long afterwards. and ultimately the interference of the surgeon is required. From this it may seem that I am averse to leaving such cases entirely to nature; occasionally the surgeon cannot do otherwise, and sometimes, even with all his care, matter will burst forth when he does not expect it. If an abscess in the perinium, for example, be left for a day or two, under the supposition that the delay will be advantageous, even though it may be intended to use the knife to open it, the practitioner is often amazed to find that the matter has, in the lapse of four-and twenty hours, made an exit through the skin or mucous membrane in the vicinity of the anus, —yet it seldom happens in such instances, that the knife is not ultimately required.

"The period for giving exit to matter will vary according to circumstances. Thus, if it be extensively diffused, as in phlegmonous erysipelas, where it may separate the skin from the textures underneath to a most injurious degree, the sooner a free vent to the matter is given, so much the better; and here, too, if the knife be used, there seems an additional advantage, as has been pointed out by Mr. Lawrence, in the loss of blood occasioned by the division of engorged vessels: again, if the pressure of matter seems to produce great pain, or if its continued presence is likely in any way to occasion additional harm, such as by bursting into any cavity, laying bare a portion of bone, or of a large vessel; or if from being under an aponeurosis, there is greater chance of its being diffused beneath that membrane than making its way to the surface, then it should be permitted to escape through an incision made at an early period. However, excepting under these or other equally pressing circumstances, I must declare myself an advocate for delay in opening abscesses. Besides the hope, though it may be but slender, of absorption occurring, I am of opinion that in ordinary abscess, a bubo, for instance, if an opening is not made until the matter has approached near to the surface, the subsequent progress of the case is much more rapid and satisfactory, provided that a proper opening be made. I have seen a good deal of the practice of making early openings, and have invariably observed that more pain was thereby induced, and seemingly too, an additional amount of suppuration, whilst the after-treatment has been remarkably tedious. My opinion has already been stated in a preceding page, that in some instances the occurrence of suppuration actually produces an amelioration of many of the distressing accompaniments of inflamation; but if, before the process has gone almost to its full extent, any incision be made into the affected parts, the injury thereby inflicted upon them will, to say the least of it, be a most untimely interference. That in all instances of suppurative inflammation this is not the case, may be readily admitted; and the examples cited above, to inculcate the necessity for an early opening, will show that I entertain many exceptions; but in the case of bubo in the groin, or other such abscess, I am decidedly inclined to delay, until the parts over the matter become much thinner than they may have been at first, or, in other words, until the fluid has come nearer to the surface, and perhaps actually threatens to burst forth."

In the treatment of Erysipelas, while our author holds constitutional remedies to be necessary, he has but "little faith in those which are said by some to check the progress of the disease in a sudden, specific and mysterious manner," as the Nitrate of Silver locally applied. He says:

"It cannot be denied that erythema and simple erysipelas have disappeared under the use of such means, but it does not follow that the cure is to be attributed to them; doubtless in many instances the circumstance has been overlooked, that these conditions, so soon as they are developed, have a natural tendency to disappear, and it is probably in such cases that the marvelous effects of these measures have been evinced. Treat two cases, or any equal numbers of a similar affection, one with nitrate of silver, another with such remedies as have been recommended for inflammation, and in all probability the results will be nearly alike under either method; in one, however, there will be no pretence of suddenly arresting the progress of the disease, which must of necessity run a certain course, whilst with the other there is an affectation of power on the part of the surgeon, which, in reality, he does not possess. Similar objections may be raised to the use of flour or chalk, or smearing the part with mercurial ointment; the two former seem occasionally to have a cooling effect, and moreover they absorb the moisture, which is exuded in the form of cuticular bullæ or vessicles, in many cases of erysipelas; and the latter, with most equivocal merits, has the disadvantage of being a most filthy mode of treatment. I imagine that the heating influence of flour may probably be greater than the cooling, and can see little benefit in the absorbing effect of this material, of chalk, or of any other similar remedy When fluid is actually present, and when such means is used, a dirty cake is generally formed on the surface, which, if not soon removed, often proves a source of irritation; the secretion, forming a vesicle, becomes semi-purulent, and thus the supposed remedy becomes an evil. In excoriations of surface, slight inflammations from certain apparent sources of irritation, such as may be noticed in very fat children, or even grown up persons, or from urine coming constantly in contact with the skin during any period of life, there may be no particular objection to the remedies in question, although in all such cases attention to cleanliness, and a proper change of dressings may be equally conducive to good; but in erysipelas they are often, I imagine, worse than useless, and in the cases where they are said to have been useful it may be doubted if the disease has really been present, or if so, whether it has not got well in spite of such applications. Very recently it had been the custom with some to apply collodion in such cases, which, it is said, has acted with admirable effect. In all probability the above remarks are equally applicable here."

The treatment of Aneurism by pressure is favorably considered, as harmless when judiciously applied, affording the patient a chance of recovery without an operation and not diminishing his chances in the last resort. Prof. F. remarks:

"Since these remarks were published in the last edition of this work, the treatment of aneurism by compression has attracted further attention, and the results, in the hands of the Dublin surgeons especially, have placed the success of the practice beyond doubt. Much credit is due to Dr. Bellingham, of Dublin, for the attention he has paid to this interesting subject, and Mr. Tufnell, of the same city, is not less worthy of praise. The latter gentleman has published a most admirable treatise upon it, and Dr. Bellingham's most recent views have been published in a short paper on the Treatment of Popliteal Aneurism by Compression, in the thirty-fourth volume of the Transactions of the Royal Medical and Chirurgical Society, 1851. From this paper it appears that 36 cases of external aneurism had been treated in this manner in Dublin, during the preceding seven years. To use Dr. Bellingham's own words, 'in 29 of these a cure was effected by compression; of the remaining 7 cases the artery was tied in 2, the patients recovering. In 1, pressure was discontinued, the aneurism subsequently diminished in size, and the patient had the perfect use of the limb for three years, when symptoms of aneurism of the aorta supervened and compelled him to give up his employment. In 2, the limb was amputated, the patients recovering; and in the remaining 2, death occurred, in one from pulmonary disease, in the other from a severe attack of erysipelas; but, in both, the local disease was very nearly cured, the aneurismal sacs being almost completely filled by fibrine deposited in concentric layers.' In not one of these cases can it be said that evil resulted from pressure. If erysipelas was the result of the interference of the surgeon, it was more likely the sequence of the galvano-puncture, which was resorted to in this instance in conjunction with compression, than from the pressure. These 36 cases were treated by 21 different surgeons, and both numbers give fair practical inference of what may be expected from a continuance of the practice among surgeons generally. The method has been tried frequently by others than the Dublin surgeons, but as yet we have no data from their cases to form any positive conclusions regarding it. Many instances have been mentioned to me wherein it has failed, but I have heard of many more in which it has been successful; and if Dr. Bellingham's Table—which, as we learn from the Dublin Medical Press, for December 3, 1851, has since been increased to 62 cases—be contrasted with some of those showing the results of ligature of the femoral artery in the hands of the various surgeons, the balance seems greatly in favor of compression. It has been ascertained by Dr. Norris, that of 188 cases, in which the operation was performed, 46 died, the majority of this number being from causes directly attributable to the use of the knife. In 6 of the cases of recovery in the above list, amputation of the limb was required. Of 119 cases of popliteal aneurism collected in a tabular form by Dr. Crisp, 16 died, and in 6 of those wherein recovery took place, amputation was resorted to. So far as our comparatively limited experience in the method by pressure, as followed by the Dublin surgeons, will enable us to form an estimate of its value, it seems in many respects, if not in all, preferable to that by deligation of the main artery; and there seems these great advantages in it, that if it does not act satisfactorily, the Hunterian operation may still be resorted to, with as much probability of success; a ever, while by its application none of those formidable dangers are incurred, which are the well known consequences of the application of the ligature. The difficulties and immediate dangers of a cutting operation are avoided. It may seem strange to make use of such language in the present day, as applicable to ligature of the superficial femoral artery—for that I assume to be the vessel meant by the unfortunately vague term of "femoral;" nevertheless, when it is known that great difficulties have been experienced in such a proceeding, even by hospital surgeons and teachers of surgery, and that the accompanying great vein has been wounded by different operators, the facts cannot be overlooked. Mr. Syme states, in the Edinburgh Monthly Journal of Medical Science for November, 1851, that he has tied the superficial femoral artery twenty times without a fatal issue, and with perfect success, and both he and his patients may be congratulated on such satisfactory results; but the Tables above referred to show no such average success: and when it is borne in mind that the attempt at cure by pressure does not preclude the resource of the ligature, it seems to me that, with the ample evidence before us of the great success which has attended the modern practice by this means, the surgeon should undoubtedly give it trial, ere he resorts to the knife.—Unquestionably the evil result of the Hunterian operation, in regard to ligature of the superficial femoral artery, has in many instances resulted from the defective or injurious style of operation, but the same may be said of certain examples where pressure has failed. Granting both of these statements to be correct, it cannot be overlooked that ligature of the superficial femoral artery, done to the perfection of human skill, has nevertheless been followed with the worst possible results. I have seen Mr Syme perform the operation repeatedly with admirable skill and precision in all points, and the results have been all that could be desired; but I have seen many others, and among them I may name the late Mr. Liston, perform the same operation, with an equal amount of tact and judgment, yet the results have been very different. With pressure, surgery has still further resources, but with the ligature the fate of the case is, for a time, placed almost, if not quite, beyond human power; and doubtless the surgeons of Dublin who have resorted to this practice (many of whom stand among the highest of those who have graced the annals of the profession) have duly considered all these points."

On the subject of removing malignant tumors, our author has the following observations:

"My own experience coincides entirely with that of every unprejudiced observer, that when malignant growths are removed with the knife their return is but too likely; nevertheless, as excision gives the only chance of security —a point on which most parties seem to agree—an operation should always be resorted to, provided the knife can be carried beyond the supposed limits of the disease; and, moreover, I deem it one of the duties of the practitioner to urge the patient to submit to such a proceeding. Many imagine that one time will be as good as another for such a purpose, and most will be pleased to put off the evil day; but if our professional education does not tell us how to cure such diseases by less harsh measures, it enables us, at all events, to foresee the threatened danger, and to warn the patient of it at an early period.

"When the growth is supposed to be non-malignant, there is less imperative necessity for interference. Thus in a fatty tumour, or one of an encysted character, the decision may be almost allowed to rest with the patient. The latter form is frequently met with on the scalp, and the fatty often acquires an immense size, so as to produce marked disfiguration, and perhaps inconvenience, both from its bulk and weight. If, however, the patient is willing to put up with such conditions, it is not incumbent on the surgeon to press an operation, as he should in an instance of malignant affection.

"In all cases of a doubtful nature, if the circumstances are otherwise favorable, I beleive that the surgeon best does his duty who recommends an operation. In inculcating this practice, I by no means wish it understood that the knife is to be the immediate expedient; on the contrary, in the early stages of tumours, whenever such measures can be resorted to as may be supposed to cause absorption, they should have a fair trial; but when such plans fail, as they will almost invariably do, and if there is reason to suppose that the further continuance of the disease will be detrimental to health, I deem the practitioner highly culpable who still tampers with it, and persists in placing faith in either local applications or constitutional remedies.

"Under the supposition that the vessels in a part where tumour first begins are preternaturally excited, it is customary to apply leeches in the neighbourhood; counter-irritation is afterwards resorted to, on similar views to those which lead to the employment of such a remedy in deep-seated inflammation. The plan of tying one or more of the enlarged arteries leading to the disease, as in the case of bronchocele, and thereby directly cutting off some of its channels of supply, has also been put into execution, probably on similar principles.

"The various local means which have been used on these occasions, are much the same as those already referred to in describing the treatment of inflammation and some of its consequences, and I need not, therefore enumerate them again. Iodine is the remedy in which most faith has been placed for the last twenty or thirty years; but I am free to confess, and deem it my duty to do so, that I have never in one single instance seen an organized adventitious growth removed by it. I have already stated my conviction that large swellings of serum, lymph, blood and pus, may gradually disappear,—be absorbed as we say in common language,—and firmly believe that on many occasions iodine, used internally, but particularly externally, produces more decided effects in some of these instances, than any other means we know of. Hydrocele, hydrophthalmia, effusions into joints, cysts (though rarely), enlargements of glands, such as the lymphatic or the thyroid, even gelatinous alterations of synovial membranes, will all disappear under the use of iodine; but I may challenge any one to produce a single case where an exostosis, a scirrhus, a medullary sarcoma or a fatty tumour, has been removed by its influence. I am myself a great admirer of iodine in its different forms, and often wonder whether the remedy has not suffered in character from the overweening confidence of some of the zealous advocates for its supposed extraordinary powers; in numerous instances where improvement has taken place during its use, I fear that, as in the case of many other so called specifics, there has been too little note taken of those changes, both local and constitutional, which are the results of time and other circumstances."

We have thus presented our readers with a few extracts from this excellent treatise, rather than written a review of it; but from these they will be able to form their own judgments as to the manner in which the author handles his subject. If the impression made by them is not eminently favorable, we beg them to believe that it is because the extracts are too short and are read under the disadvantage of being disconnected. It is with the most entire conviction of the value of the volume and of its perfect adaptation to the wants of the student and practitioner, that we heartily commend it to our professional brethren, of whatever age or amount of experience.

